# 6-Bromo-2-(4-nitro­phen­oxy)-3-(1-phenyl­ethyl)-3,4-dihydro-1,3,2-benzoxaza­phosphinine 2-oxide

**DOI:** 10.1107/S1600536809040379

**Published:** 2009-10-10

**Authors:** V. H. H. Surendra Babu, M. Krishnaiah, K. Srinivasulu, C. Naga Raju, B. Sreedhar

**Affiliations:** a Department of Physics, S.V. University, Tirupati 517 502, India; b Department of Materials Science and Chemical Engineering, Shizuoka University, Hamamatsu, Shizuoka 432-8561, Japan; c Department of Chemistry, S.V. University, Tirupati 517 502, India; d Laboratory of X-ray Crystallography, Indian Institute of Chemical Technology, Hyderabad 500 007, India

## Abstract

In the title compound, C_21_H_18_BrN_2_O_5_P, the six-membered oxaza­phosphinine ring is in a twist-boat conformation. One of the phosphoryl O atoms is in an equatorial configuation while the other is axial with respect to the oxaza­phosphinine ring. The mean planes of the benzene ring to which the nitro group is attached and the phenyl ring form a dihedral angle of 83.5 (1)°. In the crystal structure, weak inter­molecular C—H⋯O hydrogen bonds link the mol­ecules into chains along [100].

## Related literature

For background information on organophospho­rus heterocyclic compounds containing O and N in the six membered ring, see: Srinivasulu *et al.* (2008 ▶); Hill (1975 ▶); Reddy *et al.* (2004 ▶); Prasad *et al.* (2006 ▶); Sosnovsky & Paul (1983 ▶). For related structures, see: Krishnaiah *et al.* (2007 ▶); Pattabhi (1975 ▶); Radha Krishna *et al.* (2007 ▶); Symes *et al.* (1988 ▶); Hay & Mackay (1979 ▶); Kant *et al.* (2009 ▶); Selladurai *et al.* (1989 ▶).
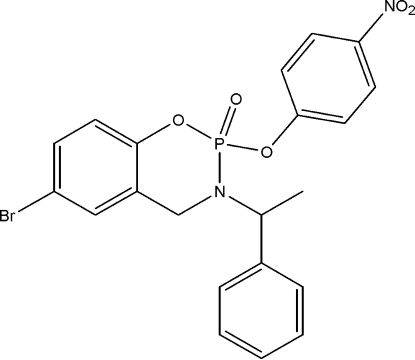


## Experimental

### 

#### Crystal data

C_21_H_18_BrN_2_O_5_P
*M* *_r_* = 489.24
Triclinic, 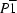

*a* = 6.9038 (6) Å
*b* = 12.0229 (11) Å
*c* = 14.0667 (13) Å
α = 111.154 (1)°
β = 97.905 (2)°
γ = 104.359 (2)°
*V* = 1021.05 (16) Å^3^
*Z* = 2
Mo *K*α radiation
μ = 2.13 mm^−1^
*T* = 294 K
0.30 × 0.28 × 0.10 mm

#### Data collection

Siemens SMART CCD area-detector diffractometer
Absorption correction: multi-scan (*SADABS*; Sheldrick, 2004 ▶) *T*_min_ = 0.533, *T*_max_ = 0.808
11716 measured reflections
4932 independent reflections
3958 reflections with *I* > 2σ(*I*)
*R*_int_ = 0.026

#### Refinement

*R*[*F* ^2^ > 2σ(*F* ^2^)] = 0.034
*wR*(*F* ^2^) = 0.092
*S* = 1.04
4932 reflections
271 parameters
H-atom parameters constrained
Δρ_max_ = 0.55 e Å^−3^
Δρ_min_ = −0.30 e Å^−3^

### 

Data collection: *SMART* (Bruker, 2001 ▶); cell refinement: *SAINT* (Bruker, 2002 ▶); data reduction: *SAINT*; program(s) used to solve structure: *SHELXS97* (Sheldrick, 2008 ▶); program(s) used to refine structure: *SHELXL97* (Sheldrick, 2008 ▶); molecular graphics: *ZORTEPII* (Zsolnai, 1998 ▶); software used to prepare material for publication: *enCIFer* (Allen *et al.*, 2004 ▶) and *PARST* (Nardelli, 1995 ▶).

## Supplementary Material

Crystal structure: contains datablocks global, I. DOI: 10.1107/S1600536809040379/lh2902sup1.cif

Structure factors: contains datablocks I. DOI: 10.1107/S1600536809040379/lh2902Isup2.hkl

Additional supplementary materials:  crystallographic information; 3D view; checkCIF report

## Figures and Tables

**Table 1:** Hydrogen-bond geometry (Å, °)

*D*—H⋯*A*	*D*—H	H⋯*A*	*D*⋯*A*	*D*—H⋯*A*
C9—H9*B*⋯O5^i^	0.97	2.49	3.352 (3)	148
C19—H19⋯O5^i^	0.93	2.50	3.404 (3)	163

Symmetry code: (i) 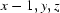.

**Fig. 1.**
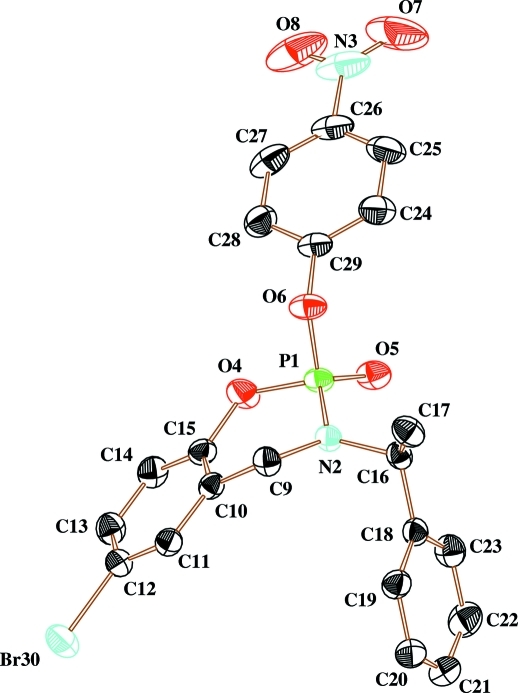
The molecular structure of the title compound. Displacement ellipsoids are drawn at the 40% probability level.

**Fig. 2.**
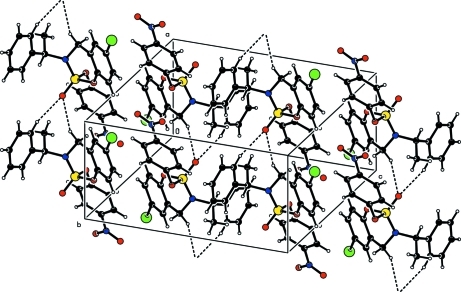
Part of the crystal structure of (I) with hydrogen bonds shown as dashed lines.

## supplementary crystallographic information

### Comment

Organophosphorus heterocyclic compounds containing O and N in the six membered ring have gained much attention ever since cyclophosphamide was discovered as an anti-cancer drug (Prasad *et al.*, 2006). Compounds of this class have high anti-tumor activity (Sosnovsky & Paul, 1983), significant bioactivity (Reddy *et al.*, 2004) and medicinal properties (Hill *et al.*, 1975). In this aspect, the title compound (I) possesses antifungal activity against *Aspergillus niger* and *Alternaria alternata*, anti bacterial aganist Gram Positive *Bacillus subtilis* and Gram negative *Escherichia coli* and also insecticidal activity against *Scirpophaga incertulas* (Srinivasulu *et al.*, 2008). These characteristics has motivated us to study the influence of the substituents on the conformation and molecular geometry of the heterocyclic ring in this type of compound.

In the crystal structure (I), the oxazaphosphinine ring adopts a twist boat conformation, with atoms C9/C10/C15/O4 coplanar and the atoms P1 and N2 are displaced in same direction by -0.562 (1) and -0.854 (2)Å respectively. When the substituents at P and N in oxazaphosphinine ring are methoxy phenyl and chloro phenyl, chlorophenoxy and chloro fluorophenyl the conformations are boat and screw boat(Radha Krishna *et al.*, 2007; Krishnaiah *et al.*, 2007). In the present study, the steric and electronic effects of the subsitutents change the conformation of the oxazaphosphinine ring to twist boat and this may be due to nitrophenoxy ring attached to the P atom and phenylethyl substituent at the N atom. The nitrophenoxy and phenylethyl rings are at axially and equatorially orientated with dihedral angles of 27.2 (1)° and 71.0 (1)° to the mean plane of heterocyclic ring.

The P=O(2) distance of 1.456 (2)Å is in good agreement with the values in related structures (Kant *et al.*, 2009; Krishnaiah *et al.*, 2007). The P—N [1.621 (2) Å], N—C [1.471 (2) Å] bond distances and P—N—C [119.0 (1)°] bond angle are agreeable with related structures in the literature (Symes *et al.*, 1988; Selladurai *et al.*, 1989). The dihedral angle between the nitro group and attached benzene ring is 8.2 (3)°. The O7—N3—O8 bond angle [125.(3)°] and average N—O bond length [1.226 (4) Å] are in agreement with the values in the related structures (Hay *et al.*, 1979; Pattabhi *et al.*, 1975). The C—Br bond length [1.895 (2) Å] is in good agreement with the value reported by Radha Krishna *et al.* (2007). In the crystal structure, molecules are linked by weak intermolecular C—H···O hydrogen bonds (see Table 1 and Fig. 2)

### Experimental

4-Nitrophenyl phosphorodichloridate 0.51 g(2.0 mmole)in dry toluene(10 ml) was added dropwise to a stirred solution of 2-[(1-phenylethylamino)methyl] -4-bromophenol 0.61 g (2.0 mmole) and triethylamine 0.40 g (4.0 mmole)in 20 ml of dry toluene at 273K  over 20 minutes. After the completion of the addition, the reaction temperature was slowly raised to 328–333K and was maintained at this temperature for 5 h. Progress of the reaction was monitored by TLC using hexane-ethyl acetate (3:1)as mobile phase on silica gel (adsorbent). Upon separation of the triethylamine hydrochloride by filtration and evaporation of the filtrate under reduced pressure, a solid residue was obtained. The residue was washed with water and diffraction quality crystal were grown by slow evaporation of a solution of the title compound in methanol.

### Refinement

H-atoms bound to carbon were positioned geometrically and refined using a riding model with d(C—H) = 0.93 Å, *U*_iso_=1.2_eq_ (C) for aromatic, C—H = 0.980Å *U*_iso_=1.2_eq_ (C) for methine, 0.97 Å, *U*_iso_ = 1.2_eq_ (C) for CH_2_ group and 0.96 Å, *U*_iso_ = 1.5_eq_ (C) for methyl H atoms.

### Crystal data

**Table:**

C_21_H_18_BrN_2_O_5_P	*Z* = 2
*M**_r_* = 489.24	*F*(000) = 496
Triclinic, *P*1	*D*_x_ = 1.591 Mg m^−^^3^*D*_m_ = 1.590 Mg m^−^^3^*D*_m_ measured by not measured
Hall symbol: -P 1	Mo *K*α radiation, λ = 0.71073 Å
*a* = 6.9038 (6) Å	Cell parameters from 4932 reflections
*b* = 12.0229 (11) Å	θ = 1.6–28.0°
*c* = 14.0667 (13) Å	µ = 2.13 mm^−^^1^
α = 111.154 (1)°	*T* = 294 K
β = 97.905 (2)°	Plate, colorless
γ = 104.359 (2)°	0.30 × 0.28 × 0.10 mm
*V* = 1021.05 (16) Å^3^	

### Data collection

**Table:**

Siemens SMART CCD area-detector diffractometer	4932 independent reflections
Radiation source: fine-focus sealed tube	3958 reflections with *I* > 2σ(*I*)
graphite	*R*_int_ = 0.026
ω–2θ scans	θ_max_ = 28.0°, θ_min_ = 1.6°
Absorption correction: multi-scan (*SADABS*; Sheldrick, 2004)	*h* = −9→9
*T*_min_ = 0.533, *T*_max_ = 0.808	*k* = −15→15
11716 measured reflections	*l* = −18→18

### Refinement

**Table:**

Refinement on *F*^2^	Primary atom site location: structure-invariant direct methods
Least-squares matrix: full	Secondary atom site location: difference Fourier map
*R*[*F*^2^ > 2σ(*F*^2^)] = 0.034	Hydrogen site location: inferred from neighbouring sites
*wR*(*F*^2^) = 0.092	H-atom parameters constrained
*S* = 1.04	*w* = 1/[σ^2^(*F*_o_^2^) + (0.0447*P*)^2^ + 0.2933*P*] where *P* = (*F*_o_^2^ + 2*F*_c_^2^)/3
4932 reflections	(Δ/σ)_max_ = 0.001
271 parameters	Δρ_max_ = 0.55 e Å^−^^3^
0 restraints	Δρ_min_ = −0.30 e Å^−^^3^

### Special details

**Table:**

Geometry. All e.s.d.'s (except the e.s.d. in the dihedral angle between two l.s. planes) are estimated using the full covariance matrix. The cell e.s.d.'s are taken into account individually in the estimation of e.s.d.'s in distances, angles and torsion angles; correlations between e.s.d.'s in cell parameters are only used when they are defined by crystal symmetry. An approximate (isotropic) treatment of cell e.s.d.'s is used for estimating e.s.d.'s involving l.s. planes.
Refinement. Refinement of *F*^2^ against ALL reflections. The weighted *R*-factor *wR* and goodness of fit *S* are based on *F*^2^, conventional *R*-factors *R* are based on *F*, with *F* set to zero for negative *F*^2^. The threshold expression of *F*^2^ > σ(*F*^2^) is used only for calculating *R*-factors(gt) *etc*. and is not relevant to the choice of reflections for refinement. *R*-factors based on *F*^2^ are statistically about twice as large as those based on *F*, and *R*- factors based on ALL data will be even larger. Weighted least-squares planes through the starred atoms (Nardelli, Musatti, Domiano & Andreetti Ric.Sci.(1965),15(II—A),807). Equation of the plane: m1**X*+m2*Y+m3**Z*=dPlane 1 m1 = -0.25244(0.00099) m2 = 0.85342(0.00085) m3 = -0.45601(0.00139) D = 1.39325(0.02640) Atom d s d/s (d/s)**2 C9 * -0.0053 0.0023 - 2.319 5.378 C10 * 0.0097 0.0021 4.554 20.741 C15 * -0.0113 0.0023 - 5.002 25.020 O4 * 0.0035 0.0018 1.974 3.897 P1 - 0.5610 0.0006 - 934.361 873029.938 N2 - 0.8542 0.0018 - 476.107 226678.125 ============ Sum((d/s)**2) for starred atoms 55.036 Chi-squared at 95% for 1 degrees of freedom: 3.84 The group of atoms deviates significantly from planarityPlane 2 m1 = -0.23579(0.00097) m2 = 0.85905(0.00049) m3 = -0.45436(0.00086) D = 1.43988(0.01431) Atom d s d/s (d/s)**2 C10 * -0.0012 0.0021 - 0.562 0.316 C11 * 0.0016 0.0023 0.711 0.506 C12 * 0.0007 0.0023 0.310 0.096 C13 * -0.0040 0.0025 - 1.585 2.513 C14 * 0.0042 0.0025 1.674 2.801 C15 * -0.0012 0.0023 - 0.527 0.277 ============ Sum((d/s)**2) for starred atoms 6.510 Chi-squared at 95% for 3 degrees of freedom: 7.81 The group of atoms does not deviate significantly from planarityPlane 3 m1 = 0.13603(0.00097) m2 = -0.10179(0.00086) m3 = -0.98546(0.00016) D = -18.25778(0.00363) Atom d s d/s (d/s)**2 C18 * 0.0049 0.0018 2.741 7.515 C19 * -0.0094 0.0022 - 4.340 18.833 C20 * 0.0040 0.0023 1.737 3.018 C21 * 0.0058 0.0024 2.373 5.630 C22 * -0.0103 0.0027 - 3.832 14.682 C23 * 0.0009 0.0023 0.392 0.153 ============ Sum((d/s)**2) for starred atoms 49.831 Chi-squared at 95% for 3 degrees of freedom: 7.81 The group of atoms deviates significantly from planarityPlane 4 m1 = -0.47634(0.00103) m2 = 0.87824(0.00057) m3 = -0.04237(0.00125) D = 8.88495(0.02266) Atom d s d/s (d/s)**2 C24 * 0.0086 0.0028 3.048 9.293 C25 * 0.0050 0.0031 1.626 2.644 C26 * -0.0119 0.0029 - 4.059 16.478 C27 * 0.0074 0.0034 2.161 4.672 C28 * 0.0089 0.0030 2.953 8.717 C29 * -0.0105 0.0024 - 4.389 19.263 ============ Sum((d/s)**2) for starred atoms 61.067 Chi-squared at 95% for 3 degrees of freedom: 7.81 The group of atoms deviates significantly from planarityDihedral angles formed by LSQ-planes Plane - plane angle (s.u.) angle (s.u.) 1 2 1.01 (0.08) 178.99 (0.08) 1 3 70.84 (0.10) 109.16 (0.10) 1 4 27.24 (0.10) 152.76 (0.10) 2 3 70.84 (0.07) 109.16 (0.07) 2 4 27.62 (0.08) 152.38 (0.08) 3 4 83.54 (0.10) 96.46 (0.10) Weighted least-squares planes through the starred atoms (Nardelli, Musatti, Domiano & Andreetti Ric.Sci.(1965),15(II—A),807). Equation of the plane: m1**X*+m2*Y+m3**Z*=dPlane 1 m1 = -0.47634(0.00105) m2 = 0.87824(0.00057) m3 = -0.04237(0.00118) D = 8.88495(0.02071) Atom d s d/s (d/s)**2 C24 * 0.0086 0.0028 3.048 9.293 C25 * 0.0050 0.0031 1.626 2.644 C26 * -0.0119 0.0029 - 4.059 16.478 C27 * 0.0074 0.0034 2.161 4.672 C28 * 0.0089 0.0030 2.953 8.717 C29 * -0.0105 0.0024 - 4.389 19.263 ============ Sum((d/s)**2) for starred atoms 61.067 Chi-squared at 95% for 3 degrees of freedom: 7.81 The group of atoms deviates significantly from planarityPlane 2 m1 = 0.39868(0.00762) m2 = -0.90292(0.00337) m3 = 0.16059(0.00247) D = -7.68046(0.09646) Atom d s d/s (d/s)**2 O7 * 0.0000 0.0038 0.000 0.000 N3 * 0.0000 0.0039 0.000 0.000 O8 * 0.0000 0.0039 0.000 0.000 ============ Sum((d/s)**2) for starred atoms 0.000 Dihedral angles formed by LSQ-planes Plane - plane angle (s.u.) angle (s.u.) 1 2 8.23 (0.27) 171.77 (0.27)

### Fractional atomic coordinates and isotropic or equivalent isotropic displacement parameters (Å^2^)

**Table:**

	*x*	*y*	*z*	*U*_iso_*/*U*_eq_	
Br30	0.07372 (3)	0.95218 (2)	0.846811 (18)	0.05590 (10)	
P1	0.98210 (7)	1.40317 (5)	1.22196 (4)	0.03470 (12)	
N2	0.7703 (2)	1.37669 (15)	1.25914 (12)	0.0344 (3)	
O4	0.9135 (2)	1.32659 (15)	1.09802 (12)	0.0500 (4)	
C12	0.3366 (3)	1.07127 (19)	0.92609 (16)	0.0394 (4)	
O5	1.1552 (2)	1.38127 (14)	1.27575 (13)	0.0463 (3)	
O8	1.9001 (4)	1.7330 (3)	1.1441 (3)	0.1177 (12)	
O6	1.0436 (2)	1.54558 (14)	1.23196 (13)	0.0468 (4)	
C16	0.7768 (3)	1.40186 (18)	1.37146 (14)	0.0350 (4)	
H16	0.9224	1.4276	1.4081	0.042*	
C19	0.4550 (3)	1.2403 (2)	1.37139 (17)	0.0430 (5)	
H19	0.3747	1.2885	1.3605	0.052*	
C9	0.5758 (3)	1.35586 (19)	1.18729 (15)	0.0372 (4)	
H9A	0.5784	1.4328	1.1789	0.045*	
H9B	0.4609	1.3335	1.2169	0.045*	
C11	0.3542 (3)	1.16589 (19)	1.02215 (15)	0.0372 (4)	
H11	0.2373	1.1719	1.0468	0.045*	
C10	0.5470 (3)	1.25190 (18)	1.08172 (14)	0.0332 (4)	
C15	0.7171 (3)	1.23950 (19)	1.04172 (15)	0.0372 (4)	
C18	0.6676 (3)	1.28190 (18)	1.38117 (14)	0.0357 (4)	
C13	0.5082 (3)	1.0600 (2)	0.88753 (17)	0.0454 (5)	
H13	0.4939	0.9954	0.8230	0.055*	
C21	0.4794 (4)	1.0567 (2)	1.39599 (19)	0.0578 (6)	
H21	0.4166	0.9812	1.4000	0.069*	
C17	0.7001 (3)	1.5121 (2)	1.42262 (18)	0.0473 (5)	
H17A	0.7766	1.5840	1.4130	0.071*	
H17B	0.7194	1.5322	1.4965	0.071*	
H17C	0.5560	1.4897	1.3905	0.071*	
C29	1.2448 (3)	1.60445 (19)	1.22977 (18)	0.0423 (5)	
C24	1.3853 (4)	1.6834 (2)	1.3241 (2)	0.0554 (6)	
H24	1.3462	1.6988	1.3871	0.066*	
C23	0.7834 (3)	1.2087 (2)	1.39997 (18)	0.0472 (5)	
H23	0.9252	1.2349	1.4071	0.057*	
C28	1.2949 (4)	1.5824 (2)	1.1350 (2)	0.0541 (6)	
H28	1.1961	1.5305	1.0720	0.065*	
C14	0.7011 (3)	1.1457 (2)	0.94568 (16)	0.0440 (5)	
H14	0.8177	1.1402	0.9205	0.053*	
C26	1.6379 (4)	1.7154 (2)	1.2300 (2)	0.0575 (6)	
C20	0.3630 (4)	1.1278 (2)	1.37790 (18)	0.0517 (5)	
H20	0.2211	1.1002	1.3699	0.062*	
C27	1.4966 (4)	1.6394 (3)	1.1355 (2)	0.0629 (7)	
H27	1.5352	1.6262	1.0725	0.076*	
C22	0.6907 (4)	1.0980 (2)	1.4082 (2)	0.0586 (6)	
H22	0.7705	1.0510	1.4220	0.070*	
C25	1.5863 (4)	1.7398 (2)	1.3240 (2)	0.0636 (7)	
H25	1.6844	1.7934	1.3870	0.076*	
N3	1.8545 (4)	1.7694 (3)	1.2296 (3)	0.0879 (10)	
O7	1.9752 (4)	1.8438 (3)	1.3132 (3)	0.1317 (13)	

### Atomic displacement parameters (Å^2^)

**Table:**

	*U*^11^	*U*^22^	*U*^33^	*U*^12^	*U*^13^	*U*^23^
Br30	0.04266 (14)	0.05503 (16)	0.05015 (15)	0.00431 (10)	0.00283 (10)	0.01044 (11)
P1	0.0238 (2)	0.0376 (3)	0.0450 (3)	0.00870 (19)	0.01247 (19)	0.0190 (2)
N2	0.0214 (7)	0.0446 (9)	0.0360 (8)	0.0080 (6)	0.0084 (6)	0.0167 (7)
O4	0.0316 (7)	0.0606 (10)	0.0450 (8)	0.0013 (7)	0.0178 (6)	0.0135 (7)
C12	0.0350 (10)	0.0412 (10)	0.0388 (10)	0.0083 (8)	0.0045 (8)	0.0173 (8)
O5	0.0285 (7)	0.0523 (9)	0.0669 (10)	0.0149 (6)	0.0140 (6)	0.0323 (8)
O8	0.0703 (15)	0.158 (3)	0.214 (3)	0.0556 (17)	0.084 (2)	0.145 (3)
O6	0.0325 (7)	0.0431 (8)	0.0727 (10)	0.0116 (6)	0.0196 (7)	0.0305 (8)
C16	0.0276 (8)	0.0402 (10)	0.0329 (9)	0.0092 (7)	0.0062 (7)	0.0119 (8)
C19	0.0358 (10)	0.0526 (12)	0.0453 (11)	0.0125 (9)	0.0127 (9)	0.0255 (10)
C9	0.0268 (9)	0.0470 (11)	0.0387 (10)	0.0143 (8)	0.0098 (7)	0.0165 (9)
C11	0.0310 (9)	0.0457 (11)	0.0391 (10)	0.0129 (8)	0.0105 (8)	0.0211 (9)
C10	0.0307 (9)	0.0404 (10)	0.0349 (9)	0.0138 (8)	0.0112 (7)	0.0200 (8)
C15	0.0319 (9)	0.0426 (10)	0.0402 (10)	0.0100 (8)	0.0132 (8)	0.0205 (8)
C18	0.0340 (9)	0.0407 (10)	0.0305 (9)	0.0102 (8)	0.0083 (7)	0.0136 (8)
C13	0.0479 (12)	0.0480 (12)	0.0385 (10)	0.0172 (10)	0.0135 (9)	0.0135 (9)
C21	0.0679 (16)	0.0510 (13)	0.0523 (13)	0.0058 (12)	0.0102 (12)	0.0293 (11)
C17	0.0433 (11)	0.0420 (11)	0.0464 (11)	0.0120 (9)	0.0116 (9)	0.0079 (9)
C29	0.0345 (10)	0.0379 (10)	0.0631 (13)	0.0115 (8)	0.0177 (9)	0.0283 (10)
C24	0.0531 (13)	0.0461 (12)	0.0603 (14)	0.0068 (10)	0.0198 (11)	0.0187 (11)
C23	0.0405 (11)	0.0531 (13)	0.0490 (12)	0.0181 (10)	0.0101 (9)	0.0206 (10)
C28	0.0462 (12)	0.0665 (15)	0.0577 (13)	0.0148 (11)	0.0149 (10)	0.0361 (12)
C14	0.0392 (10)	0.0555 (13)	0.0424 (11)	0.0187 (9)	0.0195 (9)	0.0202 (10)
C26	0.0377 (11)	0.0520 (13)	0.102 (2)	0.0128 (10)	0.0223 (13)	0.0517 (15)
C20	0.0419 (11)	0.0600 (14)	0.0493 (12)	0.0030 (10)	0.0104 (10)	0.0274 (11)
C27	0.0604 (15)	0.0811 (18)	0.0832 (19)	0.0313 (14)	0.0404 (15)	0.0598 (16)
C22	0.0678 (16)	0.0556 (14)	0.0646 (15)	0.0277 (12)	0.0126 (13)	0.0345 (12)
C25	0.0462 (13)	0.0489 (13)	0.0815 (18)	−0.0027 (11)	0.0049 (12)	0.0271 (13)
N3	0.0474 (14)	0.0891 (19)	0.171 (3)	0.0237 (14)	0.0422 (18)	0.096 (2)
O7	0.0450 (13)	0.114 (2)	0.219 (4)	−0.0130 (14)	0.0127 (18)	0.083 (2)

### Geometric parameters (Å, °)

**Table:**

Br30—C12	1.895 (2)	C13—C14	1.384 (3)
P1—O5	1.4564 (15)	C13—H13	0.9300
P1—O4	1.5841 (16)	C21—C20	1.371 (4)
P1—O6	1.6065 (15)	C21—C22	1.384 (4)
P1—N2	1.6161 (15)	C21—H21	0.9300
N2—C9	1.471 (2)	C17—H17A	0.9600
N2—C16	1.490 (2)	C17—H17B	0.9600
O4—C15	1.401 (2)	C17—H17C	0.9600
C12—C11	1.383 (3)	C29—C24	1.372 (3)
C12—C13	1.385 (3)	C29—C28	1.372 (3)
O8—N3	1.236 (4)	C24—C25	1.385 (3)
O6—C29	1.404 (2)	C24—H24	0.9300
C16—C18	1.516 (3)	C23—C22	1.380 (3)
C16—C17	1.523 (3)	C23—H23	0.9300
C16—H16	0.9800	C28—C27	1.389 (3)
C19—C20	1.386 (3)	C28—H28	0.9300
C19—C18	1.398 (3)	C14—H14	0.9300
C19—H19	0.9300	C26—C25	1.366 (4)
C9—C10	1.504 (3)	C26—C27	1.370 (4)
C9—H9A	0.9700	C26—N3	1.477 (3)
C9—H9B	0.9700	C20—H20	0.9300
C11—C10	1.389 (3)	C27—H27	0.9300
C11—H11	0.9300	C22—H22	0.9300
C10—C15	1.387 (3)	C25—H25	0.9300
C15—C14	1.381 (3)	N3—O7	1.216 (5)
C18—C23	1.394 (3)		
			
O5—P1—O4	115.12 (9)	C12—C13—H13	120.2
O5—P1—O6	110.99 (9)	C20—C21—C22	119.7 (2)
O4—P1—O6	101.10 (9)	C20—C21—H21	120.2
O5—P1—N2	116.93 (9)	C22—C21—H21	120.2
O4—P1—N2	104.48 (8)	C16—C17—H17A	109.5
O6—P1—N2	106.72 (8)	C16—C17—H17B	109.5
C9—N2—C16	119.46 (14)	H17A—C17—H17B	109.5
C9—N2—P1	118.71 (13)	C16—C17—H17C	109.5
C16—N2—P1	120.36 (12)	H17A—C17—H17C	109.5
C15—O4—P1	124.46 (12)	H17B—C17—H17C	109.5
C11—C12—C13	121.31 (19)	C24—C29—C28	122.2 (2)
C11—C12—Br30	119.49 (15)	C24—C29—O6	118.0 (2)
C13—C12—Br30	119.19 (16)	C28—C29—O6	119.8 (2)
C29—O6—P1	118.91 (12)	C29—C24—C25	119.0 (2)
N2—C16—C18	110.44 (15)	C29—C24—H24	120.5
N2—C16—C17	111.07 (16)	C25—C24—H24	120.5
C18—C16—C17	114.62 (16)	C22—C23—C18	121.0 (2)
N2—C16—H16	106.7	C22—C23—H23	119.5
C18—C16—H16	106.7	C18—C23—H23	119.5
C17—C16—H16	106.7	C29—C28—C27	118.5 (2)
C20—C19—C18	120.4 (2)	C29—C28—H28	120.8
C20—C19—H19	119.8	C27—C28—H28	120.8
C18—C19—H19	119.8	C15—C14—C13	118.72 (18)
N2—C9—C10	110.08 (15)	C15—C14—H14	120.6
N2—C9—H9A	109.6	C13—C14—H14	120.6
C10—C9—H9A	109.6	C25—C26—C27	122.3 (2)
N2—C9—H9B	109.6	C25—C26—N3	119.2 (3)
C10—C9—H9B	109.6	C27—C26—N3	118.5 (3)
H9A—C9—H9B	108.2	C21—C20—C19	120.6 (2)
C12—C11—C10	119.80 (18)	C21—C20—H20	119.7
C12—C11—H11	120.1	C19—C20—H20	119.7
C10—C11—H11	120.1	C26—C27—C28	119.1 (2)
C15—C10—C11	118.12 (18)	C26—C27—H27	120.5
C15—C10—C9	119.75 (17)	C28—C27—H27	120.5
C11—C10—C9	122.13 (16)	C23—C22—C21	120.2 (2)
C14—C15—C10	122.55 (19)	C23—C22—H22	119.9
C14—C15—O4	117.58 (17)	C21—C22—H22	119.9
C10—C15—O4	119.84 (18)	C26—C25—C24	118.9 (3)
C23—C18—C19	118.15 (19)	C26—C25—H25	120.5
C23—C18—C16	118.77 (18)	C24—C25—H25	120.5
C19—C18—C16	123.08 (17)	O7—N3—O8	125.0 (3)
C14—C13—C12	119.50 (19)	O7—N3—C26	117.8 (4)
C14—C13—H13	120.2	O8—N3—C26	117.2 (3)
			
O5—P1—N2—C9	−159.82 (14)	N2—C16—C18—C23	92.9 (2)
O4—P1—N2—C9	−31.29 (17)	C17—C16—C18—C23	−140.77 (19)
O6—P1—N2—C9	75.29 (16)	N2—C16—C18—C19	−86.7 (2)
O5—P1—N2—C16	34.08 (18)	C17—C16—C18—C19	39.7 (3)
O4—P1—N2—C16	162.61 (15)	C11—C12—C13—C14	−0.6 (3)
O6—P1—N2—C16	−90.81 (15)	Br30—C12—C13—C14	−179.15 (16)
O5—P1—O4—C15	119.48 (17)	P1—O6—C29—C24	−98.4 (2)
O6—P1—O4—C15	−120.83 (17)	P1—O6—C29—C28	81.9 (2)
N2—P1—O4—C15	−10.13 (19)	C28—C29—C24—C25	−2.1 (4)
O5—P1—O6—C29	36.11 (19)	O6—C29—C24—C25	178.2 (2)
O4—P1—O6—C29	−86.48 (17)	C19—C18—C23—C22	0.3 (3)
N2—P1—O6—C29	164.56 (16)	C16—C18—C23—C22	−179.2 (2)
C9—N2—C16—C18	74.1 (2)	C24—C29—C28—C27	2.0 (3)
P1—N2—C16—C18	−119.90 (15)	O6—C29—C28—C27	−178.4 (2)
C9—N2—C16—C17	−54.2 (2)	C10—C15—C14—C13	−0.6 (3)
P1—N2—C16—C17	111.78 (17)	O4—C15—C14—C13	−178.71 (19)
C16—N2—C9—C10	−139.77 (17)	C12—C13—C14—C15	0.8 (3)
P1—N2—C9—C10	54.0 (2)	C22—C21—C20—C19	0.2 (4)
C13—C12—C11—C10	0.0 (3)	C18—C19—C20—C21	1.2 (3)
Br30—C12—C11—C10	178.63 (14)	C25—C26—C27—C28	−1.7 (4)
C12—C11—C10—C15	0.2 (3)	N3—C26—C27—C28	176.5 (2)
C12—C11—C10—C9	−179.31 (18)	C29—C28—C27—C26	−0.1 (4)
N2—C9—C10—C15	−36.3 (2)	C18—C23—C22—C21	1.1 (4)
N2—C9—C10—C11	143.17 (17)	C20—C21—C22—C23	−1.4 (4)
C11—C10—C15—C14	0.1 (3)	C27—C26—C25—C24	1.6 (4)
C9—C10—C15—C14	179.63 (19)	N3—C26—C25—C24	−176.6 (2)
C11—C10—C15—O4	178.15 (17)	C29—C24—C25—C26	0.3 (4)
C9—C10—C15—O4	−2.3 (3)	C25—C26—N3—O7	−7.0 (4)
P1—O4—C15—C14	−154.46 (17)	C27—C26—N3—O7	174.8 (3)
P1—O4—C15—C10	27.4 (3)	C25—C26—N3—O8	171.5 (3)
C20—C19—C18—C23	−1.5 (3)	C27—C26—N3—O8	−6.8 (3)
C20—C19—C18—C16	178.04 (19)		

### Hydrogen-bond geometry (Å, °)

**Table:**

*D*—H···*A*	*D*—H	H···*A*	*D*···*A*	*D*—H···*A*
C9—H9B···O5^i^	0.97	2.49	3.352 (3)	148
C19—H19···O5^i^	0.93	2.50	3.404 (3)	163

Symmetry codes: (i) *x*−1, *y*, *z*.
